# Postoperative Pain and Functional Limitations After Corneal Cross-Linking for Keratoconus: A Patient-Reported Outcome Study

**DOI:** 10.3390/life16040694

**Published:** 2026-04-21

**Authors:** Ruta Jaruseviciene, Gintare Kirkickaite, Saulius Galgauskas

**Affiliations:** Institute of Health Sciences, Faculty of Medicine, Vilnius University, M. K. Ciurlionio Str. 21, LT-03101 Vilnius, Lithuania; gintare.kirkickaite@mf.stud.vu.lt (G.K.); saulius.galgauskas@mf.vu.lt (S.G.)

**Keywords:** keratoconus, corneal cross-linking, postoperative pain, patient-reported outcomes, quality of life, functional limitations

## Abstract

Background: Keratoconus is a progressive corneal condition that leads to visual impairment and is primarily managed with corneal cross-linking (CXL), a procedure designed to halt its progression. However, while the clinical outcomes of CXL are well-documented, its impact on patient well-being, including postoperative pain and recovery, remains underexplored. This study aimed to evaluate postoperative pain, functional limitations, visual recovery, and patient-reported outcomes following corneal cross-linking (CXL) for keratoconus. Methods: A structured survey was conducted among 31 patients who underwent CXL for keratoconus. The survey assessed postoperative pain using a Numeric Rating Scale (NRS) and collected information on visual recovery, functional limitations, and the socioeconomic effects of the procedure. Clinical data, including best-corrected visual acuity (BCVA) and keratometry, were also recorded before and after CXL to evaluate the procedure’s efficacy. Results: The study found that postoperative pain was moderate, with a mean pain score of 6.06 ± 1.82, typically lasting 1–3 days. Nearly 54.8% of patients reported significant disruption to daily activities, including missing work or studies, and 77.4% experienced some degree of functional limitation. A statistically significant correlation was observed between postoperative pain intensity and quality-of-life impairment (Spearman’s ρ = 0.503, *p* = 0.004). Despite these challenges, 93.5% of participants reported improvement in vision, with most recovering within a week. Objective clinical data supported the effectiveness of the procedure. Conclusions: Corneal cross-linking is associated with favorable clinical outcomes; however, the early postoperative period is characterized by moderate pain and functional limitations, highlighting the importance of patient-centered care.

## 1. Introduction

### 1.1. Background

Keratoconus is a bilateral and asymmetric condition characterized by progressive thinning and steepening of the cornea, which ultimately leads to irregular astigmatism and decreased visual acuity [1]. Although historically described as a non-inflammatory disease, recent findings suggest the involvement of inflammatory cytokines, oxidative stress, and corneal extracellular matrix degradation. Elevated levels of matrix metallopeptidase 9 (MMP-9), interleukin 6 (IL-6), and tumor necrosis factor-alpha (TNF-α), along with oxidative stress markers, have been identified in affected corneas [2].

The condition commonly begins during adolescence, progresses over 10–20 years, and stabilizes in adulthood [3]. Its prevalence varies across geographic regions and is influenced by genetic, environmental and ethnic factors. UV exposure, atopy, and eye rubbing are among the recognized contributors. Individuals residing in sunnier regions may face a higher risk due to increased ultraviolet light exposure and associated oxidative stress [3]. However, geographic variation in keratoconus prevalence cannot be explained by latitude alone, as regional differences are also influenced by genetic, environmental, and behavioral factors. For example, while higher prevalence has been reported in regions such as India and the Middle East, other areas at similar latitudes may demonstrate different epidemiological patterns [3,4].

### 1.2. Treatment Modalities

Early identification of keratoconus is critical for timely interventions, most notably corneal crosslinking (CXL), which can forestall more invasive measures. The standard Dresden protocol involves removing the epithelium followed by riboflavin application and subsequent ultraviolet A (UV-A) irradiation [4]. While effective, this procedure carries risks such as ocular pain, corneal melting, and infections [5].

To minimize these adverse effects, researchers introduced a variation of the Dresden protocol that keeps the epithelium intact, thereby reducing the likelihood of complications. However, evidence from randomized controlled trials in pediatric cohorts indicates that the standard Dresden method yields superior visual and keratometric outcomes, facilitating smoother corneal remodeling [6]. Both the accelerated CXL and conventional methods demonstrate efficacy, with the transepithelial technique lagging behind [6].

### 1.3. Economic and Socioeconomic Implications

Keratoconus presents a considerable economic burden attributed to its early onset and detrimental impact on visual quality of life [7]. Historically, treatments such as spectacles, contact lenses, intrastromal corneal ring segments (ICRSs), and keratoplasty have proven costly, uncomfortable, and oftentimes ineffective [4]. Currently, corneal crosslinking is favored for its minimally invasive nature. One-year follow-up studies post-CXL demonstrate improvements in topographic measurements, visual acuity, and refractive stability, with outcomes stabilizing within six months [4].

Despite being the favored treatment for keratoconus, CXL has notable socioeconomic implications. Many studies indicate that postoperative pain can be intense even with effective pain management, particularly in younger patients, indicating a notable socioeconomic impact. This impact can be seen in the potential loss of productivity due to the need for extended recovery periods, increased healthcare costs associated with pain management, and the psychological toll on the patient [8,9].

Additionally, the use of anti-inflammatory drops is essential in mitigating the risk of postoperative infections. Although the incidence of infectious keratitis following epithelium-off CXL is relatively low, bacterial pathogens are the most frequently encountered, usually manifesting around 4.8 days post-surgery [10]. Notably, certain factors such as the use of steroids, bandage contact lenses applied immediately after the procedure, and a history of atopic or herpetic conditions may heighten the risk of infection. Implementing thorough postoperative care is vital for these patients, including timely prophylactic antiviral therapy when necessary [10]. However, adherence to postoperative pharmacological therapy may also impose an additional burden on patients due to cost and treatment complexity.

Therefore, the aim of this study was to investigate postoperative pain, functional limitations, and patient-reported outcomes following corneal cross-linking for keratoconus, while also evaluating associated clinical outcomes. However, despite well-established clinical outcomes, there remains a lack of detailed evidence on patient-reported postoperative experience, particularly regarding pain, functional limitations, and short-term quality-of-life impact.

## 2. Materials and Methods

### 2.1. Study Design

A structured observational, questionnaire-based study was conducted to evaluate patient-reported outcomes following corneal cross-linking (CXL). The primary objective of the study was to assess postoperative pain, functional limitations, visual recovery, and patient-reported outcomes after CXL. The study was conducted between June and October 2025 after approval by the Vilnius Regional Biomedical Research Ethics Committee.

### 2.2. Participants and Inclusion Criteria

The study included 31 participants (52 eyes). Inclusion criteria were:

1. Confirmed diagnosis of keratoconus;
2. Treatment with epithelium-off CXL;
3. Age ≥ 18 years.

No restrictions were applied regarding disease stage or bilateral involvement. No specific exclusion criteria were applied apart from the absence of keratoconus diagnosis or incomplete clinical data. No specific exclusion criteria were applied in order to reflect real-world clinical practice. Patients with incomplete clinical or questionnaire data were excluded from the analysis.

### 2.3. Data Collection

A structured questionnaire was administered to all participants within one month after the corneal cross-linking procedure during a routine postoperative visit. The questionnaire used in this study was not formally validated. The questionnaire used in this study was not formally validated (see Supplementary File S1). The questionnaire required approximately 10–15 min to complete and was administered once for each participant. It consisted of self-reported items designed to capture a detailed profile of the postoperative experience, focusing on pain intensity, ocular symptoms, medication use, visual recovery, functional limitations, and the impact of the procedure on daily activities.

Pain Assessment: Postoperative pain intensity was measured using a Numeric Rating Scale (NRS), where 0 represented no pain and 10 indicated the most severe pain imaginable. Patients were asked to specify the peak pain level, its duration (several hours, 1–2 days, 2–3 days, or more than 3 days), and whether additional medical attention was sought.

Medication Use: Participants reported all medications used for postoperative relief, including oral painkillers (e.g., ibuprofen, paracetamol), topical antibiotic eye drops, corticosteroid drops, NSAIDs, and lubricating artificial tears. The survey also identified whether patients required repeated dosing or prolonged therapy.

Ocular Symptoms: Patients indicated the presence and frequency of common postoperative symptoms such as burning, irritation, dryness, tearing, photophobia, foreign body sensation, and blurred vision. They were also encouraged to freely describe any additional sensations not listed in the questionnaire.

Visual Recovery: Respondents were asked to report the timeline for return of functional vision, including whether recovery occurred within a few days, within one week, within two weeks, or longer. Patients were also asked to assess perceived changes in visual quality following the procedure.

Impact on Daily Functioning and Socioeconomic Factors: The questionnaire captured the extent to which postoperative discomfort affected daily activities, such as work, studying, household tasks, and leisure activities. Participants indicated whether they missed work or school, required assistance, or avoided routine activities. Additional questions explored the economic burden related to time off work and medication use.

Clinical Data Collection: Objective clinical data, including best-corrected visual acuity (BCVA) and keratometry values (Kmax, Kmin), were obtained before and after CXL to evaluate the structural and functional effectiveness of the procedure. To minimize potential bias, all participants completed the same structured questionnaire under standardized conditions during routine postoperative visits. Patients were recruited consecutively to reduce selection bias. The questionnaire was administered within one month after the procedure to limit recall bias. Patient-reported outcomes were complemented with objective clinical data.

### 2.4. CXL Procedure

Corneal cross-linking was performed using the standard epithelium-off (Dresden) protocol. After topical anesthesia, the central 8–9 mm of the corneal epithelium was mechanically removed. Riboflavin 0.1% solution was instilled every 2–3 min for 30 min to achieve adequate stromal saturation. This was followed by ultraviolet-A (UV-A) irradiation at 370 nm with an irradiance of 3 mW/cm^2^ for 30 min, with continued riboflavin instillation at regular intervals.

At the end of the procedure, a bandage contact lens was applied and maintained until complete epithelial healing. Postoperatively, all patients received a standardized treatment regimen consisting of topical antibiotic eye drops, topical corticosteroids, and lubricating artificial tears. Oral analgesics (e.g., ibuprofen or paracetamol) were prescribed as needed for pain control. Patients were followed up routinely until complete epithelial healing and stabilization of symptoms.

### 2.5. Statistical Analysis

Statistical analysis was carried out using *IBM SPSS Statistics version 29.0* (IBM Corp., Armonk, NY, USA). Absolute and relative frequencies were generated for categorical variables, while continuous variables were summarized using means and standard deviations. The distribution of continuous variables was assessed. As assumptions of normality were not met, nonparametric methods were applied. Continuous variables are presented as mean ± standard deviation or median (interquartile range, IQR), where appropriate.

The association between postoperative pain intensity and patient-reported quality of life impact was examined using the Spearman rank correlation coefficient. Differences in pain scores across functional impairment categories were evaluated using the Kruskal–Wallis test, whereas comparisons of visual outcomes between patient groups were conducted using the Mann–Whitney U test. Results were analysed separately for right and left eyes to improve clarity; however, this approach does not fully account for within-subject correlation.

All statistical tests were two-tailed, and statistical significance was defined as *p* < 0.05. The study was reported in accordance with the STROBE guidelines for observational studies.

## 3. Results

### 3.1. Demographic and Baseline Clinical Characteristics

All participants enrolled in the study (*N* = 31) were diagnosed with keratoconus and underwent corneal collagen cross-linking as part of their clinical management. The majority of participants were men (71%), with 29% being women. The mean age at the time of CXL was 27.85 ± 7.26 years, indicating that most participants were young adults at an active stage of their professional or educational life.

Most patients (67.7%) underwent bilateral CXL, while unilateral treatment (OD or OS) was performed in 32.3% of cases. All patients had clinically confirmed bilateral keratoconus.

On average, the duration from diagnosis to the CXL procedure was 3.71 ± 6.35 years, suggesting that intervention occurred relatively early in the disease course.

Baseline clinical evaluation demonstrated reduced best-corrected visual acuity (BCVA) and increased keratometric values, consistent with progressive keratoconus. Prior to CXL, baseline clinical characteristics are summarized in Table 1. Keratometry revealed pronounced corneal steepening, with mean Kmax values of 50.32 ± 7.43 D (OD) and 48.71 ± 5.08 D (OS).

Baseline demographic and clinical characteristics of the study population are summarized in Table 1.

### 3.2. Postoperative Pain Intensity and Duration

Pain intensity following epithelium-off CXL procedure was moderate to severe. The mean reported pain score was 6.06 ± 1.82 on a 0–10 numerical scale, with the majority of patients reporting scores of 6–8. The mode value was 6, confirming that moderate pain was typical postoperative experience.

**Figure 1:**
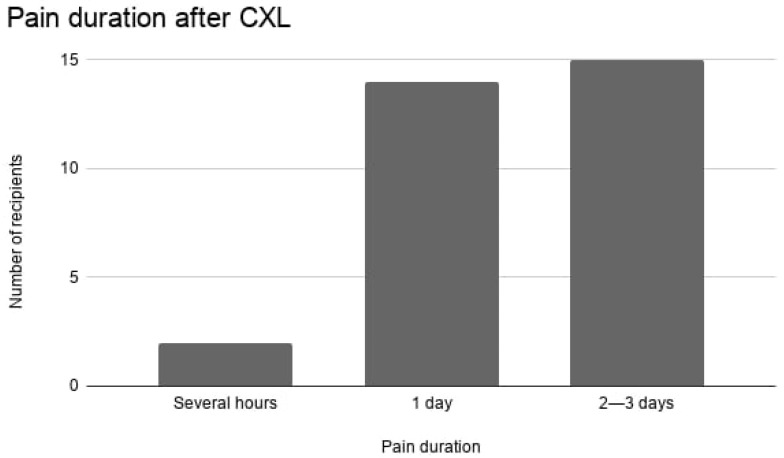
Distribution of postoperative pain duration following epithelium-off corneal cross-linking. Most patients experienced pain lasting one to three days, consistent with corneal epithelial healing.

Regarding duration, nearly all patients experienced pain lasting at least one full day. Most (48.4%) reported pain persisting for 2–3 days, while 45.2% experienced pain lasting a single day. Only 6.5% indicated that pain lasted only several hours. No cases reported pain persisting beyond 3 days. Postoperative pain intensity and duration are summarized in Table 2 and illustrated in Figure 1.

These findings align with expected epithelial healing timelines following the epithelium-off technique.

### 3.3. Postoperative Ocular Symptoms, Medication Use, and Quality-of-Life Impact

The majority of patients (83.9%) experienced at least one unpleasant ocular symptom during the early postoperative period. However, the distribution of individual symptoms appeared heterogeneous and may reflect limitations of the questionnaire rather than true symptom prevalence. Tearing was the most frequently reported symptom (12.9%), followed by photophobia, dryness, burning, and foreign-body sensations (each 9.7%), while blurred vision was reported by only 6.5% of patients. These findings indicate that transient and generally mild ocular symptoms are common during the early post-procedural period, though they rarely present as isolated or severe issues. Patients were allowed to report multiple symptoms; therefore, percentages reflect the proportion of patients reporting each symptom.

Medication use was nearly universal. All patients reported the use of oral analgesics (e.g., ibuprofen, paracetamol), topical antibiotic eye drops, and topical corticosteroids, reflecting the standard postoperative treatment regimen prescribed after CXL and does not necessarily reflect individual symptom-driven medication needs. Artificial tears were less commonly used, with 19.4% of respondents reporting their use.

**Figure 2:**
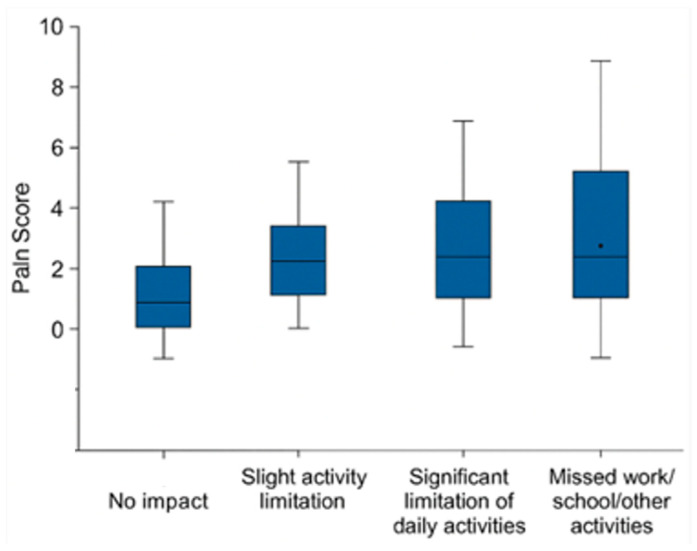
Boxplots illustrate pain scores (0–10) across four categories of self-reported quality-of-life impact (*n* = 31): no impact, mild limitation, significant limitation, and inability to attend work, school, or other activities. The box represents the interquartile range, the line indicates the median, and the dot represents the mean.

Despite the transient nature of symptoms, the procedure had a noticeable impact on daily functioning. Out of 31 respondents, the majority (54.8%) indicated that the procedure had a substantial impact, to the extent that they had to miss work, studies, or other important activities. An additional 22.6% experienced significant limitations in daily tasks, while 12.9% reported no impact on quality of life. These findings suggest that although the procedure was generally well-tolerated, more than three-quarters of participants (77.4%) experienced some degree of functional limitation during the recovery period, with over half facing major disruptions (Figure 2). Although patients reporting greater impairment tended to report higher pain scores, the Kruskal–Wallis test did not reveal statistically significant differences between groups (χ^2^(3) = 7.779, *p* = 0.051), although the result indicated a trend toward statistical significance.

A nonparametric analysis revealed a statistically significant moderate correlation between postoperative pain intensity and perceived impact on quality of life (Spearman’s ρ = 0.503, *p* = 0.004; 95% CI: 0.171 to 0.733). A trend toward differences in pain intensity across quality-of-life impact categories was observed using Kruskal–Wallis test (*p* = 0.051); however, post hoc pairwise comparisons were not statistically significant after Bonferroni correction.

Postoperative symptoms, medication use, and quality-of-life impact are summarized in Table 3.

### 3.4. Visual Recovery and Objective Clinical Outcomes

Visual recovery was rapid in most patients. Nearly half of respondents (48.4%) reported regaining functional vision within several days after the procedure, while 45.2% achieved functional visual recovery within one week. Only two patients (6.5%) required up to two weeks for visual recovery, and no patient reported prolonged visual impairment.

Subjective visual outcomes were predominantly positive. Improvement in vision was reported by 93.5% of patients, whereas only 6.5% perceived a deterioration following the procedure. The overall procedural experience was rated as either “very good” or “good” by 77.4% of respondents, indicating high patient satisfaction despite early postoperative discomfort.

Objective clinical evaluation confirmed these subjective findings. BCVA improved in both treated eyes following CXL. Mean BCVA in the right eye increased from 0.60 ± 0.30 preoperatively to 0.83 ± 0.17 postoperatively, while in the left eye it improved from 0.64 ± 0.28 to 0.87 ± 0.12. These improvements indicate a clinically meaningful gain in visual function. The improvement in BCVA before and after CXL was statistically significant according to the Wilcoxon signed-rank test (*p* < 0.05 for both eyes).

Keratometric analysis demonstrated a reduction in corneal steepening after CXL. Mean Kmax values decreased from 50.32 ± 7.43 D to 46.92 ± 5.87 D in the right eye and from 48.71 ± 5.08 D to 47.72 ± 4.54 D in the left eye. Similar changes were observed in Kmin values, reflecting biomechanical stabilization of the cornea. Changes in keratometric values before and after CXL were also evaluated using the Wilcoxon signed-rank test.

Table 4A,B summarize the improvement in best-corrected visual acuity and reduction in corneal curvature (Kmax, Kmin) in both eyes following CXL treatment. The data shows improvements in visual acuity and stabilization trends of corneal shape, confirming the effectiveness of the procedure.

## 4. Discussion

To our knowledge, few studies have evaluated patient-reported postoperative burden after CXL. The present study investigated patient-reported postoperative pain and functional limitations after epithelium-off corneal cross-linking for keratoconus. In agreement with previous reports, our findings are consistent with previous evidence suggesting that CXL is an effective intervention for improving best-corrected visual acuity (BCVA) and stabilizing corneal topography. At the same time, our results demonstrate that the early postoperative period is frequently characterized by moderate pain and a temporary but meaningful impact on daily functioning [4,8,9].

Postoperative pain intensity in this cohort was moderate to severe, with a mean score exceeding 6 on a 0–10 scale. Pain duration was limited to the first few postoperative days, most commonly lasting one to three days, which is consistent with epithelial healing following the epithelium-off technique. These results are consistent with earlier prospective and observational studies that documented peak pain scores occurring within the first postoperative days, followed by rapid resolution [8,9,11]. Soeters et al. reported a median VAS score of 6.2 (range 0 to 10), closely mirroring the pain intensity observed in our study [11]. Importantly, no cases of prolonged or chronic pain were reported in line with systematic reviews indicating that persistent pain after CXL is exceedingly rare [9]. This supports the notion that postoperative discomfort, although intense, is self-limiting and confined to the acute recovery phase.

The early postoperative period was frequently characterized by ocular pain, photophobia, and temporary visual fluctuations, consistent with previous reports emphasizing similar short-term challenges [8,11,12].

Beyond pain intensity alone, our results highlight a meaningful association between postoperative discomfort and short-term quality-of-life impairment. The observed moderate positive correlation suggests that higher pain levels are associated with greater functional limitations, including the need to miss work or studies. Although between-group differences did not reach statistical significance after correction for multiple comparisons, the observed trend indicates a clinically meaningful relationship that may become more evident in larger cohorts. These findings highlight the importance of effective pain management strategies and thorough patient counseling, particularly for young, professionally active individuals. Compared to previous studies, the pain intensity observed in our cohort was similar, although some reports describe slightly lower peak pain scores, possibly due to differences in postoperative management protocols.

Despite early postoperative discomfort, overall patient satisfaction was high. Subjective improvement in vision was reported by more than 90% of participants, and objective measurements demonstrated significant improvements in BCVA along with stabilization of or reduction in keratometric indices (Kmax and Kmin). These findings are consistent with both short- and long-term studies showing that CXL leads to sustained improvements in visual acuity and corneal biomechanics [4,13,14,15]. Previous long-term studies have demonstrated sustained improvements in visual acuity and stabilization of keratometric indices following CXL [13,14,15].

Parallel to these functional gains, keratometric indices, particularly the anterior maximum keratometry (Kmax) value, tend to decrease, reflecting a corneal flattening. Multiple cohort studies have reported Kmax reductions of approximately 1–2 diopters within the first 12 months post-treatment, with these gains remaining stable from the first to the third year postoperatively [13,14,15]. A significant long-term study covering ten years indicated that Kmax reductions reached statistical significance one year following surgery and maintained consistency throughout the decade [13]. This continuous flattening of the corneal curvature is clinically significant, as it reduces irregular astigmatism and higher-order aberrations, thereby enhancing optical regularity and correlating with the observed improvements in both UDVA and CDVA. These findings suggest that CXL may contribute to stabilization of the ectatic process associated with keratoconus; however, some variability in keratometric outcomes was observed. In particular, although mean Kmax values decreased after CXL, the changes were not entirely uniform, and median values in some cases did not show consistent improvement. This variability should be considered when interpreting the clinical effectiveness of the procedure in this cohort.

In comparison with other keratoconus management strategies, such as spectacles, contact lenses, and intracorneal ring segments, CXL primarily aims to halt disease progression rather than correct refractive error. While these alternatives may improve visual acuity, they do not address the underlying ectatic process. In contrast, CXL provides biomechanical stabilization of the cornea and may reduce the need for more invasive procedures such as keratoplasty.

In conclusion, while CXL offers significant benefits in terms of vision improvement and corneal stabilization, the procedure is associated with moderate pain and functional limitations during the recovery period. These findings suggest that pain management strategies and patient counseling regarding the recovery process could enhance the overall experience and improve patient satisfaction. Since patients still experience considerable pain and require medication postoperatively, it would be valuable to explore additional methods to further reduce discomfort while maintaining the procedure’s effectiveness. Further studies with larger sample sizes are needed to explore the relationship between postoperative pain, quality of life, and long-term outcomes in keratoconus patients undergoing CXL. These findings suggest that clinicians should provide detailed preoperative counseling regarding the expected postoperative discomfort and temporary limitations in daily functioning. These findings should be considered exploratory and hypothesis-generating. Future prospective studies using diary-based designs would provide more accurate assessment of postoperative patient experience.

## 5. Limitations

Some limitations of the present study should be acknowledged. The relatively small sample size limits statistical power, particularly for subgroup analyses, and may explain the lack of statistical significance in some comparisons despite observable trends. In addition, both eyes from some participants were included in the analysis, which may introduce within-subject dependence and limit the independence of observations. Although results were presented separately for right and left eyes, this approach does not fully eliminate this limitation. Postoperative experience was assessed retrospectively using self-reported questionnaires, which may be subject to recall bias and reporting bias. Furthermore, the questionnaire used in this study was not formally validated, which may limit comparability with findings from other studies. The study also did not include a control group or comparison with other corneal procedures, limiting the ability to determine whether the observed patient-reported outcomes are specific to CXL or comparable to other interventions. Future research with larger cohorts and prospective designs is needed to better elucidate the relationship between postoperative symptoms, quality of life, and long-term clinical outcomes following corneal cross-linking. The study did not stratify outcomes based on unilateral versus bilateral treatment, which may influence postoperative pain intensity, recovery, and functional limitations. This should be addressed in future studies. Additionally, due to the standardized postoperative treatment regimen, it was not possible to evaluate differences in pain intensity based on the type of analgesics used. Furthermore, the relatively low proportion of patients using artificial tears limited subgroup analysis. In addition, detailed information regarding the type of occupation, number of missed workdays, and specific daily activities affected (e.g., driving, screen use) was not collected, which limits a more comprehensive assessment of the socioeconomic impact. These factors may have influenced the accuracy of patient-reported outcomes and limited the ability to detect subgroup differences. In particular, recall of peak pain intensity and duration may be subject to distortion, especially given variability in treatment experience and postoperative recovery.

## 6. Conclusions

This study highlights both the clinical effectiveness of corneal cross-linking (CXL) and the patient-reported postoperative experience in individuals with keratoconus. The procedure was associated with meaningful improvements in best-corrected visual acuity and stabilization of corneal topography, confirming its therapeutic value in slowing disease progression. From the patient perspective, most participants reported improved vision and high overall satisfaction following the procedure.

At the same time, the findings emphasize that the early postoperative period is frequently accompanied by moderate ocular pain and transient symptoms such as irritation, dryness, and photophobia. These symptoms often resulted in temporary limitations in daily functioning, including missed work or studies. The observed association between pain intensity and perceived quality-of-life impairment highlights the clinical importance of addressing postoperative discomfort more effectively.

Overall, while CXL remains a cornerstone in the management of keratoconus, optimizing perioperative care and postoperative pain management may further improve patient recovery and overall treatment experience. Future studies with larger cohorts and prospective designs are needed to better understand the relationship between postoperative symptoms, functional limitations, and long-term clinical outcomes.

## Figures and Tables

**Table 1:** Baseline demographic and clinical characteristics of the study participants undergoing corneal collagen cross-linking (*n* = 31). IQR—interquartile range (25th–75th percentile).

Variable	Category	*n* (%) or Mean ± SD
Total eyes treated		52
Sex, n (%)	Female	9 (29.0%)
	Male	22 (71.0%)
Age (years)	Mean ± SD	27.85 ± 7.26
	Median (IQR)	27 (22–33)
	Range	18–44
Keratoconus laterality	Bilateral (OU)	31 (100%)
Duration from diagnosis (years)	Mean	3.71 ± 6.35
	Median (IQR)	1 (0–4)
	Range	0–22
Eye treated with CXL	OD	5 (16.1%)
	OS	5 (16.1%)
	OU	21 (67.7%)
BCVA before CXL	Mean ± SD	OD: 0.60 ± 0.30
		OS: 0.64 ± 0.28
	Median (IQR)	OD: 0.60 (0.50–0.90)
		OS: 0.70 (0.50–0.90)
Kmax before CXL (D)	Mean ± SD	OD: 50.3 ± 7.4
		OS: 48.7 ± 5.1
	Median (IQR)	OD: 49.3 (46.2–52.6)
		OS: 46.1 (44.6–52.8)
Kmin before CXL (D)	Mean ± SD	OD: 45.6 ± 5.1
		OS: 45.1 ± 3.9
	Median (IQR)	OD: 45.2 (41.9–47.2)
		OS: 44.2 (43.0–46.1)

**Table 2:** Postoperative pain intensity and duration after CXL. IQR—interquartile range (25th–75th percentile).

Variable	Category	*n* (%) or Mean ± SD
Postoperative pain score (0–10)	Mean ± SD	6.06 ± 1.82
	Median (IQR)	6 (5–7)
	Range	2–9
Pain duration	Several hours	2 (6.5%)
	1 day	14 (45.2%)
	2–3 days	15 (48.4%)

**Table 3:** Postoperative symptoms, medication use, and impact on daily functioning.

Variable	*n* (%)
Any postoperative symptom	26 (83.9%)
Tearing	4 (12.9%)
Photophobia	3 (9.7%)
Burning sensation	3 (9.7%)
Dryness	3 (9.7%)
Foreign-body sensation	3 (9.7%)
Blurred vision	2 (6.5%)
Any medication use	31 (100%)
Analgesics	31 (100%)
Antibiotic eye drops	31 (100%)
Corticosteroid eye drops	31 (100%)
Artificial tears	6 (19.4%)
Any impact on daily functioning	27 (87.1%)
No impact	4 (12.9%)
Mild limitation	3 (9.7%)
Significant limitation	7 (22.6%)
Missed work/studies	17 (54.8%)

**Table 4:** (**A**) Changes in best-corrected visual acuity (BCVA) after corneal collagen cross-linking (*n* = 31). IQR—interquartile range (25th–75th percentile). (**B**) Changes in corneal keratometry after corneal collagen cross-linking (*n* = 31). IQR—interquartile range (25th–75th percentile).

**(A)**		
**Variable**	**Category**	**Mean ± SD or Median (IQR)**
BCVA (OD)	Before CXL After CXL	0.60 ± 0.30 0.83 ± 0.17
	Median (IQR)	Before: 0.60 (0.50–0.90) After: 0.90 (0.70–0.90)
BCVA (OS)	Before CXL After CXL	0.64 ± 0.28 0.87 ± 0.12
	Median (IQR)	Before: 0.70 (0.50–0.90) After: 0.90 (0.80–0.90)
**(B)**		
**Variable**	**Category**	**Mean ± SD or Median (IQR)**
Kmax OD [D]	Before CXL After CXL	50.3 ± 7.4 46.9 ± 5.9
	Median (IQR)	Before: 49.32 (46.15–52.57) After: 48.08 (42.19–51.34)
Kmax OS [D]	Before CXL After CXL	48.7 ± 5.1 47.7 ± 4.5
	Median (IQR)	Before: 46.07 (44.57–52.77) After: 48.85 (43.40–51.47)
Kmin OD [D]	Before CXL After CXL	45.6 ± 5.1 43.2 ± 4.7
	Median (IQR)	Before: 45.24 (41.90–47.24) After: 44.25 (40.48–46.23)
Kmin OS [D]	Before CXL After CXL	45.14 ± 3.9 44.05 ± 3.3
	Median (IQR)	Before: 44.20 (43.02–46.10) After: 43.14 (41.27–46.04)

## Data Availability

The original contributions presented in this study are included in the article and Supplementary Material. Further inquiries can be directed to the corresponding author.

## Supplementary Materials

The following supporting information can be downloaded at: https://www.mdpi.com/article/10.3390/life16040694/s1, File S1: Postoperative Experience and Pain Questionnaire Following Corneal Cross-Linking.

## Abbreviations

The following abbreviations are used in this manuscript:

CXL	Corneal cross-linking
BCVA	Best-corrected visual acuity
NRS	Numeric rating scale
NSAIDs	Non-steroidal anti-inflammatory drugs
UDVA	Uncorrected distance visual acuity
CDVA	Corrected distance visual acuity
