# Supplementary material for: Postoperative Pain and Functional Limitations After Corneal Cross-Linking for Keratoconus: A Patient-Reported Outcome Study
Source: Life (Basel). 2026 Apr 21;16(4):694. doi: 10.3390/life16040694 (PMC13117727; doi:10.3390/life16040694)
Supplement: Supplementary file 1 [file life-16-00694-s001.zip › life-4227302-supplementary.pdf]

Supplementary File S1

Postoperative Experience and Pain Questionnaire Following Corneal Cross-Linking

1. Please evaluate the intensity of pain experienced after the corneal cross-linking procedure using a scale from 0 to 10 that best reflects your perceived pain level.
This is your personal perception — there are no right or wrong answers.

(Select the most appropriate option)

0 – No pain
You feel completely normal, without any discomfort or pain.
1 – Very mild pain
Barely noticeable pain. Does not interfere with daily activities.
2 – Mild pain
Slight discomfort or pain, noticeable but not limiting.
3 – Below moderate pain
Pain is noticeable but tolerable. May cause minor inconvenience.
4 – Moderate pain
Pain is clearly present and may interfere with usual activities.
5 – Moderate to strong pain
Pain is uncomfortable and more difficult to tolerate. Often requires rest or medication.
6 – Strong pain
Pain interferes with daily functioning. Medication is frequently needed.
7 – Very strong pain
Pain is intense and significantly limits activities. Nearly unbearable without medication.
8 – Extremely strong pain with severe discomfort
Pain is very intense, difficult to tolerate even with medication.
9 – Nearly unbearable pain
Very severe pain causing considerable distress. Medication provides minimal relief.
10 – Worst imaginable pain
The most severe pain imaginable. Requires immediate medical attention.

2. How long did you experience pain after the procedure?

1. Several hours
2. 1 day
3. 2–3 days
4. Longer than 3 days

3. Did you need to use medications for pain or discomfort relief?

1. Yes
2. No

4. If yes, which medications did you use?

(Multiple answers possible)

1. Oral pain medications (e.g., ibuprofen, paracetamol)
2. Antibiotic eye drops
3. Anti-inflammatory eye drops (e.g., corticosteroids)
4. Artificial tears
5. All of the above
6. Other (please specify): ____________________

5. Did postoperative pain or discomfort affect your quality of life?

1. No, everything was as usual
2. Yes, slightly limited activities
3. Yes, significantly limited daily activities
4. Yes, I had to miss work / studies / usual activities

6. Did you experience any of the following postoperative symptoms?

(Multiple answers possible)

1. Burning sensation
2. Dryness
3. Tearing
4. Photophobia (light sensitivity)
5. Foreign-body sensation
6. Blurred vision
7. All of the above
8. Other (please specify): ____________________

7. How quickly did your functional vision return after the procedure?

1. Within several days
2. Within 1 week
3. Within 2 weeks
4. Longer than 2 weeks

8. How would you rate your overall experience following the procedure?

1. Very good
2. Good
3. Moderate
4. Poor
5. Very poor

9. Do you feel that your visual acuity changed after the procedure?

1. Improved
2. Worsened

3. No change
